# Removal of Textile Dye Mixture by Fe_3_O_4_/Acrylamide/Triacryloylhexahydro Triazine Composite Hydrogel Polymer

**DOI:** 10.3390/polym17182469

**Published:** 2025-09-12

**Authors:** Sude Sena Erdağı, Can Serkan Keskin, Semra Yılmazer Keskin, Ayşe Avcı

**Affiliations:** 1 Institute of Natural Science, Sakarya University, Sakarya 54050, Türkiye; sena.erdagi@ogr.sakarya.edu.tr; 2 Faculty of Science, Department of Chemistry, Sakarya University, Sakarya 54050, Türkiye; syilmazer@sakarya.edu.tr; 3 Faculty of Engineering, Department of Food Engineering, Sakarya University, Sakarya 54050, Türkiye; aysea@sakarya.edu.tr

**Keywords:** magnetic hydrogel polymer, acrylamide, 2,4,6-triallyloxy-1,3,5-triazine, malachite green, acid violet 19

## Abstract

A swellable magnetic polymer with high removal capacity was produced. The copolymer consisting of acrylamide and 2,4,6-triallyloxy-1,3,5-triazine was synthesized via the radical polymerization method. Previously prepared magnetic Fe_3_O_4_ particles with the co-precipitation method were added during the synthesis, and then the obtained composite was hydrolyzed. The composite became a swellable hydrogel after hydrolysis. The synthesized magnetic composite hydrogel polymer was used for Malachite Green (MG) and Acid Violet 19 (AV19) binary textile dye mixture removal. A derivative method was developed to calculate the individual concentration of dyes in mixture solutions. The accuracy and precision of the developed method were examined by calculating the recovery percentage (R%) and relative standard deviation (RSD%). The highest removal percentages (~99% for MG and ~100% for AV19) were achieved at the dye mixture’s natural pH (pH 4). Antibacterial tests were examined against Gram-negative and Gram-positive bacteria, and the synthesized composite hydrogel polymer showed higher activity. The FTIR, XRD, SEM, and EDS analyses were also performed to characterize the synthesized materials.

## 1. Introduction

Cross-linked synthetic copolymers have covalently bonded three-dimensional network structures, and hydrogels are the most useful type of these polymers. They are hydrophilic, robust, have a large surface area, and can retain water with their three-dimensional structure [1]. These polymers can be used in agriculture, medicine, and wastewater treatment studies through their water retention capacity [2]. Pollutant penetration into the three-dimensional structure of the polymer provides high removal due to more interactions. Hydrogels can be used to remove both metal ions and organic contaminants [3]. Textile dyes are the major organic pollutants worldwide and can be toxic, mutagenic, and carcinogenic for aquatic life [4]. Adsorption, biodegradation, electrochemical, and catalytic methods have been used for dye removal [5]. Different sorbents have been produced for the adsorption process to obtain cheaper, practical, and highly efficient removal. Many types of hydrogels are also used in dye removal studies. Njuguna and Schönherr [6] synthesized oligoethyleneglycol dithiols cross-linked xanthan gum hydrogels to remove Gentian Violet dye. B.Tech et al. [7] used polyvinyl alcohol–glutaraldehyde cross-linked hydrogel beads to remove Congo Red dye. Ren et al. [8] produced gelatin/chitosan/β-cyclodextrin/sodium humate hydrogel for removing Methylene Blue and Acid Fuchsin dyes. Polyacrylamide-based hydrogel polymers are also used for dye removal. Lin et al. [9] prepared a polyacrylamide–chitosan hydrogel for Xylenol Orange removal. Halyal et al. [10] used polyacrylamide to remove Methylene Blue without modification. Faizan et al. [11] synthesized polyacrylamide hydrogels with sodium alginate and acrylic acid. Better removal efficiency was reported using sodium alginate–polyacrylamide–acrylic acid against Methylene Blue dye.

Recently, interest in composite polymers for removal studies has grown due to additional properties, such as high removal efficiency, a more robust polymer, and easy separation from solution. Shaki [12] synthesized a polyacrylamide–iron sulfate hybrid polymer to remove acid dyes with flocculation. Salama [13] used cellulose-grafted soy protein isolate–hydroxyapatite structure as an adsorbent for Methylene Blue dye. Deb et al. [14] produced a polyaniline functionalized ZnO nanocomposite to evaluate Eriochrome Black T anionic dye removal. One of the materials used to prepare composites is F_3_O_4_ (magnetite). It is an iron oxide with the chemical formula (Fe(III))_tet_[Fe(II)Fe(III)]_oct_O_4_ that has excellent magnetic properties [15]. These kinds of magnetic polymers have also been used for dye removal. Gao et al. [16] synthesized various Fe_3_O_4_ hyper-cross-linked polymer composites using 1,3,5-triphenylbenzene, biphenyl, triptycene, and p-terphenyl as monomers and dimethoxymethane as a crosslinker for Crystal Violet adsorption. The maximum adsorption capacity was achieved using 1,3,5-triphenylbenzene. Mu et al. [17] prepared graphene–polyaniline–Fe_3_O_4_ nanocomposites to remove Congo Red. Ali and İsmail [18] fabricated Fe_3_O_4_–polypyrrole–carbon black composite to uptake Congo Red and Methylene Blue dyes. Some studies used acrylamide and Fe_3_O_4_ polymer composite with different co-additives. Huaman et al. [19] produced Fe_3_O_4_–poly(2-hydroxyethylmethacrylateco-2-acrylamido-2-methylpropanesulfonic acid) as an adsorbent to remove Methylene Blue dye. Jiang et al. [20] synthesized Fe_3_O_4_-embedded chitosan-crosslinked-polyacrylamide to remove Sunset yellow food dye. Keskin [21] used Fe_3_O_4_–acrylamide-1,3,5-triacryloylhexahydro-1,3,5-triazin to remove Dispers Blue 56 textile dye.

The magnetic Fe_3_O_4_ particles were embedded into the acrylamide-2,3,5-triazine polymer and hydrolyzed to obtain a swellable ionic hydrogel in this study. The synthesized novel magnetic hydrogel was used to investigate the removal of the Malachite Green and Acid Violet 19 (Acid Fuschine Red) binary textile dye mixture for the first time. These dyes are frequently used in industry and endanger aquatic life with their aromatic groups. Experiments were conducted on the mixture of dye solutions because textile wastewater is generally mixed. The main problem in analyzing mixed dye solutions is the overlapping absorption bands. One of the frequently used methods for the analysis of individual components in mixtures is the derivative method. Therefore, a first-derivative method was developed to analyze dyes, and the accuracy and precision of the proposed method were demonstrated. The produced composite hydrogel polymer can be a new alternative in wastewater treatment with high removal efficiency and magnetic properties.

## 2. Materials and Methods

### 2.1. Chemicals and Reagents

High-purity (≥99) chemicals were used. Ammonium iron(III) sulfate dodecahydrate, ammonium iron(II) sulfate hexahydrate, acrylamide, 2,4,6-triallyloxy-1,3,5-triazine, dimethyl sulfoxide (DMSO), 2,2-diethoxyacetophenone (DEAP), N,N,N′,N′-tetrametyletylenediamine (TEMED), NaOH, HCl, HNO_3,_ and methanol were purchased from Merck. All the glassware was washed with diluted HNO_3_, methanol, and ultrapure water to remove metallic and organic impurities.

### 2.2. Synthesis of Fe_3_O_4_ Particles

Fe_3_O_4_ particles were synthesized using the previously described co-precipitation method with minor modifications [22,23,24]. The mixture solution (0.25 mol/L) of Fe^3+^ and Fe^2+^ was prepared from ammonium iron(III) sulfate dodecahydrate and ammonium iron(II) sulfate hexahydrate. NaOH solution (2.0 mol/L) was used as a precipitation reagent. To form Fe_3_O_4_ particles, 5.0 mL of the iron mixture solution was added dropwise into the vigorously stirred NaOH solution previously adjusted to 80 °C under reflux. After 30 min, the particles were collected with an externally applied magnet, washed with ultrapure water several times, and dried in an oven at 80 °C.

### 2.3. Synthesis of Fe_3_O_4_/Acrylamide/Triazine Composite Polymer

Synthesis was carried out using free radical polymerization [25,26]. Acrylamide as the monomer (0.5 g, ~7.0 mmol) and 2,4,6-triallyloxy-1,3,5-triazine as the crosslinker (0.5 g, ~2.0 mmol) were dissolved in 2.0 mL of DMSO by ultrasonication for five minutes. Then, 1.0 g of dry Fe_3_O_4_ was added to the mixture and ultrasonicated again. Finally, 0.1 mL of DEAP was added and mixed for ten minutes. The final mixture solution was exposed to UV light (Black Ray model B-100, UVP Inc. (Upland, CA, USA) mercury lamp, 365 nm maximum wavelength) for 60 min. The obtained composite polymer (CP) was washed with ultrapure water and methanol to remove unreacted reagents. The CP was separated by an external magnet and dried in air.

### 2.4. Hydrolysis of Fe_3_O_4_/Acrylamide/Triazine Composite Polymer

The hydrolysis solution was prepared by adding 10.0 mL TEMED to 90.0 mL of 0.1 mol/L NaOH. Dry CP was added to this solution and mixed on an orbital shaker for one day. The obtained composite hydrogel polymer (CHP) was washed with ultrapure water and methanol to remove unreacted reagents and then dried in an oven at 40 °C.

### 2.5. Dye Removal Experiments

The dry CHP was used for removal experiments. Malachite Green (MG) and Acid Violet 19 (AV19), used as target pollutant dyes, were supplied by a local textile factory. The dyes interacted with the CHP in a mixture form using the batch method with an orbital shaker (Heidolph). Many removal parameters were investigated, such as pH (2–12), interaction time (10–210 min), shaking speed (50–200 rpm), temperature (20–55 °C), dye concentration (25–400 mg/L), and CHP amount (0.05–0.5 g). All experiments were performed using 25 mL of dye solution. After the treatment, the CHP was separated from the solution using an external magnet, and the supernatant solution absorbance was measured with a Shimadzu UV-2600 model Ultraviolet–Visible spectrophotometer.

### 2.6. Development of the First Derivative Method

The first derivative method was used for individual dye concentration calculation. In this method, a graph is drawn with the first derivatives of the absorbance of the standard dye solution versus the wavenumbers. The graph has zero crossing points where the derivative value of one dye is zero or close to zero, and the other dye is higher. Calibration curves are drawn using these points. The absorbances of standard dye solutions with concentrations of 1.0–10.0 mg/L were measured between 200 and 800 nm at 0.1 nm intervals. The method’s accuracy and precision were evaluated by calculating the recovery (R%) and relative standard deviation (RSD%) values with 25 dye mixtures. The limit of detection (LOD) and limit of quantitation (LOQ) values were calculated using a 0.01 mg/L concentration of each dye (as blank) in the mixtures with the following formulas:

(1) $$LOD=\frac{3.3{sd}_{bl}}{S};\;LOQ=\frac{10{sd}_{bl}}{S}$$

where sd_bl_ is the standard deviation of the blank solution measurements, and S is the slope of the calibration curves.

### 2.7. Characterization

Field Emission–Scanning Electron Microscope (FE–SEM) (FEI–Quanta 450 FEG) images of Fe_3_O_4_ particles, CHP, and dye-treated CHP (treated with a mixture of MG and AV19 dyes) were taken at 5.13 × 10^−4^ Pa and 15.00 kV accelerating voltage. Fourier Transform Infrared (Perkin Elmer—Spectrum Two) analyses of dyes, CHP, and dye-treated CHP were performed with an ATR attachment. The materials were dried at 40 °C before the analyses. X-ray Diffraction (XRD) analysis was recorded by Rigaku D/Max 2200 equipped with graphite monochromatized Cu Kα radiation (λ = 1.5406 Å).

### 2.8. Antibacterial Activity Assays

The antibacterial activity of the CHP was determined according to the literature studies with some modifications [27,28]. *Pseudomonas aeruginosa*, *Escherichia coli* O157:H7, *Escherichia coli* type 1, *Staphylococcus aureus*, and *Listeria monocytogenes* were used as test organisms. Tryptic soy broth was used for cultivation at 37 °C for 24 h. An amount of 50 µL of each bacterial culture (108 cfu/mL) was spread onto Mueller-Hinton agar, and 0.2 mg CHP was placed. The petri dishes were incubated at 37 °C for 24 h. The inhibition zones were evaluated in millimeters. All tests were performed in four replicates.

## 3. Results and Discussion

**Figure 1:**
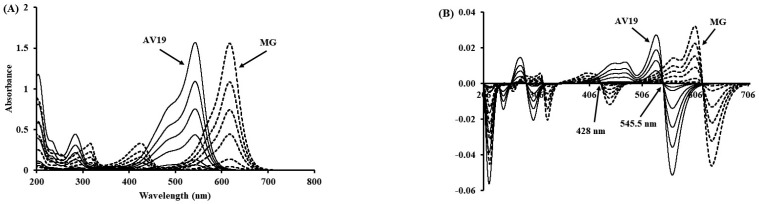
Absorbance (**A**) and first derivative (**B**) spectra of standard solutions.

A first derivative method was developed to calculate individual AV19 and MG dye concentrations in mixture solutions. The obtained absorption and first derivative spectra of standard solutions (1.0–10 mg/L) are shown in Figure 1. The calibration curve of AV19 was drawn at 428 nm (zero crossing point of MG), and MG was drawn at 545.5 nm (zero crossing point of AV19) wavelengths (Figure S1). The obtained coefficients of determination (R^2^) values were 1.0000 and 0.9996 for AV19 and MG, respectively. LOD and LOQ values are also calculated and shown in Table 1. LOQ values (<0.3) are acceptable for calculating removed dye concentrations.

The accuracy and precision of the method were examined with 25 prepared mixture solutions. The absorption and first derivative spectra of the solutions are shown in Figure S2. Table 2 shows the calculated R% and RSD% values. The mean R% is 100.84 for AV19 and 100.62 for MG. The calculated RSD% values are 2.43 and 2.40 for AV19 and MG, respectively. The first derivative method that was developed has good accuracy and precision.

### 3.1. Characterization of the Materials

**Figure 2:**
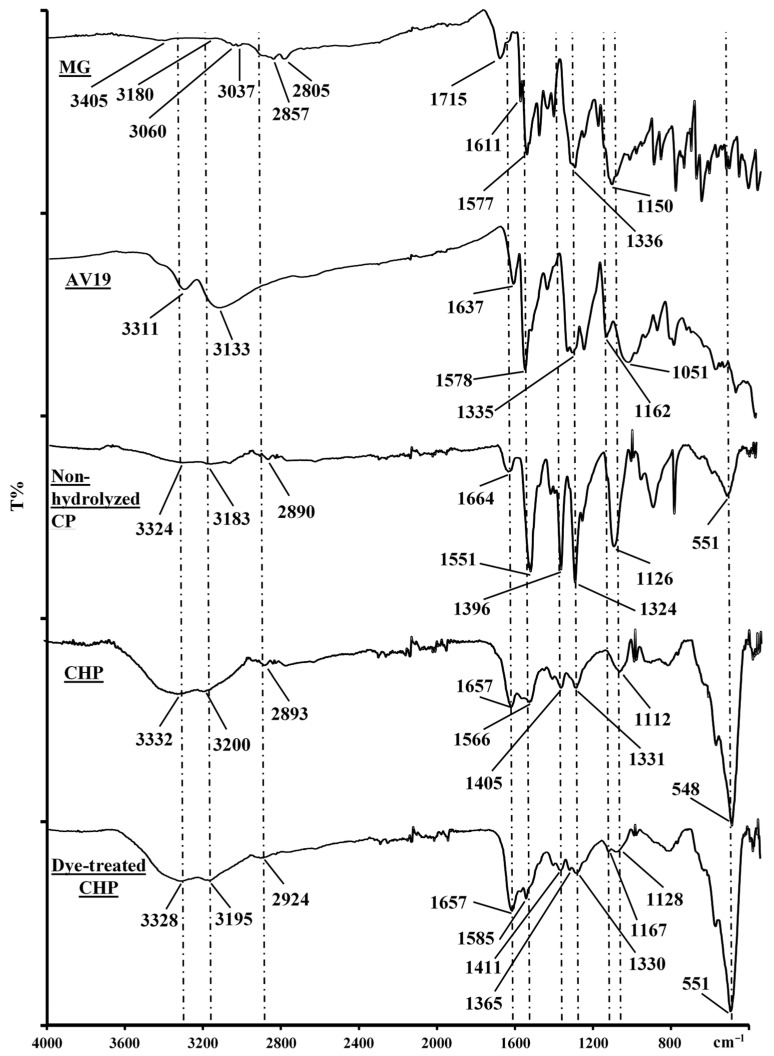
FTIR spectra of dyes and polymers.

**Figure 3:**
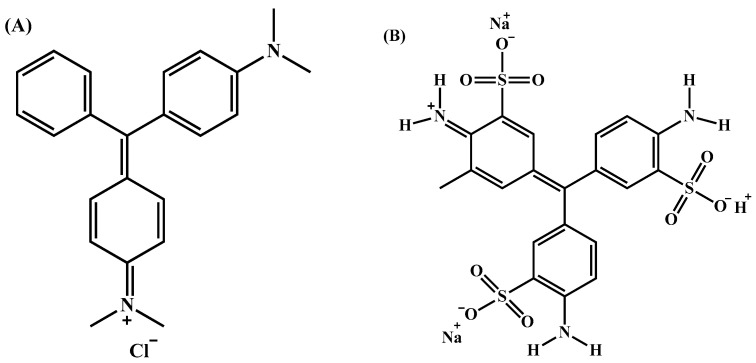
Molecular structure of MG (**A**) and AV19 (**B**).

FTIR analyses were performed to understand the interaction between dyes and CHP. The spectra of MG, AV19, non-hydrolyzed CP, CHP, and dye-treated CHP are shown in Figure 2. MG is a cationic dye with aromatic rings (Figure 3A) [29]. Therefore, the vibration peaks of the aromatic and aliphatic groups are observed in the FTIR spectrum of dyes. The peaks at 3405 cm^−1^ and 3180 cm^−1^ belong to N-H vibrations. The aromatic C-H vibration peaks appear at 3060 cm^−1^ and 3037 cm^−1^, and the aliphatics are 2857 cm^−1^ and 2805 cm^−1^. The 1715 cm^−1^ and 1611 cm^−1^ peaks belong to aliphatic and aromatic C=C vibrations [30,31]. C=N vibration peak appears at 1577 cm^−1^. The peaks at 1336 cm^−1^ and 1150 cm^−1^ are attributed to the C–N vibration [32]. AV19 also has an aromatic structure but carries both negative and positive groups in its molecular structure (Figure 3B) [33]. The 3311 cm^−1^ and 3133 cm^−1^ peaks in the FTIR spectrum belong to N–H vibrations. The aromatic C–H peaks overlapped with these peaks. The peak at 1637 cm^−1^ is attributed to the aliphatic C=C group. The C=N and aromatic C=C vibration bands overlapped and peaked at 1578 cm^−1^. The peaks of C–N groups are observed at 1335 cm^−1^ and 1162 cm^−1^. The vibrational band of the SO_3_ group appeared at 1050 cm^−1^ [34].

**Figure 4:**
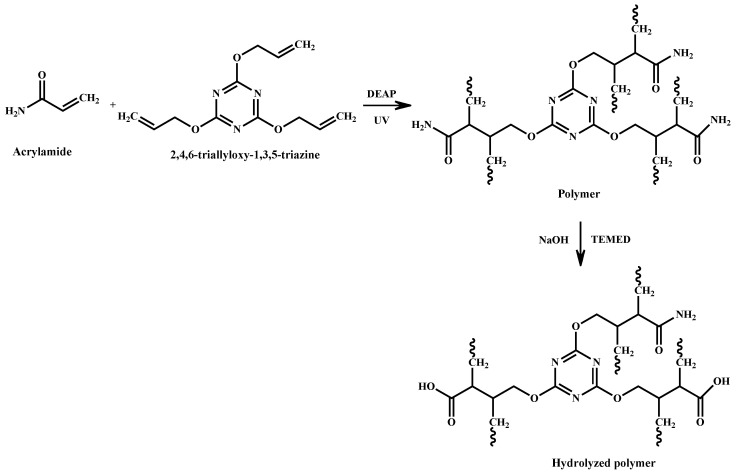
Synthesis schema of the polymer.

The synthesis schema of the polymer is shown in Figure 4. Some of the NH_2_ groups in the polymer were turned into OH groups via hydrolysis.

Similar peaks appeared in the FTIR spectra of the non-hydrolyzed CP and the CHP, except for the OH peaks. The Fe–O peak originating from the Fe_3_O_4_ particles appeared at 551 cm^−1^ in the CP and 548 cm^−1^ in the CHP [35]. The vibrations of the NH_2_ groups present at the acrylamide side of the polymer were observed at 3324 cm^−1^ and 3183 cm^−1^ in the CP spectrum. These peaks appeared at 3332 cm^−1^ and 3200 cm^−1^ and overlapped with the OH band formed after hydrolysis in the CHP spectrum. The NH_2_ group peaks in the CHP show that not all groups are hydrolyzed. The 2890 cm^−1^ and 1664 cm^−1^ peaks are attributed to aliphatic C–H and C=O vibrations in the CP spectrum. The C=N, C–N, and C–O group vibrations originating from the triazine structure appeared at 1551 cm^−1^, 1396 cm^−1^, and 1126 cm^−1^, respectively [36]. The C–N vibration in the amide group is observed at 1324 cm^−1^. Some of these peaks are shifted in the CHP. The vibration peaks of the C–H, C=O, and C=N groups appeared at 2893 cm^−1^, 1657 cm^−1^, and 1566 cm^−1^. The C–N vibration peak of triazine and amid group is formed at 1405 cm^−1^ and 1331 cm^−1^. The C–O vibrations from the carboxylate and triazine overlapped and appeared at 1112 cm^−1^. New peaks are observed in the dye-treated polymer at 1365 cm^−1^ and 1167 cm^−1^. These peaks belong to the C–N groups from the dye molecules. The vibrations of other dye groups overlapped with the polymer groups, causing slight shifts and increases in intensity.

**Figure 5:**
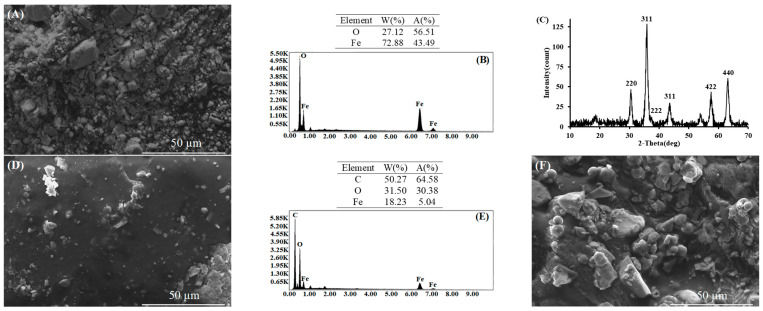
SEM images of Fe_3_O_4_ particles (**A**), EDS and XRD pattern of Fe_3_O_4_ (**B**,**C**), SEM images of CHP (**D**), EDS pattern of CHP (**E**), and SEM images of dye-treated CHP (**F**).

The photograph and magnetic behavior of CHP are shown in Figures S3 and S4. The composite material is black in color due to the embedded Fe_3_O_4_ particles. The magnetic properties of the Fe_3_O_4_ particles were also observed after polymer coating. The SEM images are shown in Figure 5.

The synthesized Fe_3_O_4_ particles are not uniform (Figure 5A) and gain sharp edges due to agglomeration. The XRD pattern of the synthesized Fe_3_O_4_ particles is shown in Figure 5C. All diffraction peaks match the characteristic diffractions of the Fe_3_O_4_ particles [37]. It can be seen in Figure 5D that Fe_3_O_4_ particles are embedded in the polymer, which is further confirmed by the EDS pattern of CHP (Figure 5E). The high-intensity C peak comes from the polymer structure. Fe_3_O_4_ peak intensities decreased because they remained under the polymer. A smooth surface was obtained by trapping the particles in the polymer. It can also be seen that some Fe_3_O_4_ particles exist on the polymer surface. The surface of CHP becomes rougher after dye interaction (Figure 5F). Spherical and differently formed dyes were collected on the surface of dye-treated CHP.

### 3.2. Effect of Operating Parameters

**Figure 6:**
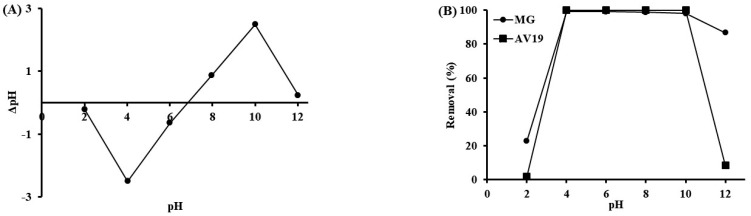
pH_pzc_ graph (**A**) and pH effect (**B**) on the removal (50 mg/L dyes, 60 min, 150 rpm shaking speed, 0.1 g of CHP, 25 °C, 25 mL of dye solutions).

The interaction between CHP and dyes mainly depends on the ionization of the functional groups via solution pH. The CP hydrolysis facilitated the dye solution’s penetration into the polymer and caused it to swell. Therefore, the dyes can interact with CHP’s inside and outside functional groups. The pH-dependent charges of the polymer can be understood by a point-of-zero charge pH (pH_pzc_) analysis (Figure 6A). The pH of the KNO_3_-CHP solution was measured at the beginning and after 24 h of shaking for pH_pzc_ determination. The found value was 6.8 from the graph drawn between the initial pH and ΔpH. The CHP is more negatively charged above pH_pzc_ and more positively charged below it. The charges on the CHP are balanced at around pH 6.8. Negative and positive charges are formed via deprotonating carboxylic acid and protonating amine groups on the polymer. pH-dependent dye removal percentages followed this result. Experiments were performed with 50 mg/L of each dye (25 mL) in 60 min at 150 rpm shaking speed, 25 °C, using 0.1 g of Fe_3_O_4_ embedded polymer. Removal efficiencies are high for both dyes between pH 4 and 10 (Figure 6B). The polymer has positive and negative charges at these pH values. The pH of the dye mixture solutions was measured as around four without any adjustment, and the highest removal efficiency (~99% for MG and ~100% for AV19) was obtained at this pH value. MG has a positive amine group in its molecular structure. AV19 has negative and positive charges via hydrolysis of –SO_3_Na and protonation of –NH_2_ groups. The negative groups of the CHP interact with the positive groups of MG at pH 4, and the dye is accumulated. Similarly, the positive and negative groups of AV19 interact with the negative and positive groups of the CHP. Removal rate is low at pH 2 (~2%) for AV19 due to competition from hydrogen ions, resulting in decreased ionization. Since ionization is very low at pH 12, the removal percentage is low (~8%). The removal efficiencies for MG were ~23% and ~87% at pH 2 and 12. The positive charge of MG is not affected by pH due to its molecular structure. However, the charges of the polymer are affected, and the removal efficiency remains low at pH 2. At pH 12, the polymer is mostly negatively charged, and the percentage of interaction with the positive groups of AV19 is still high.

**Figure 7:**
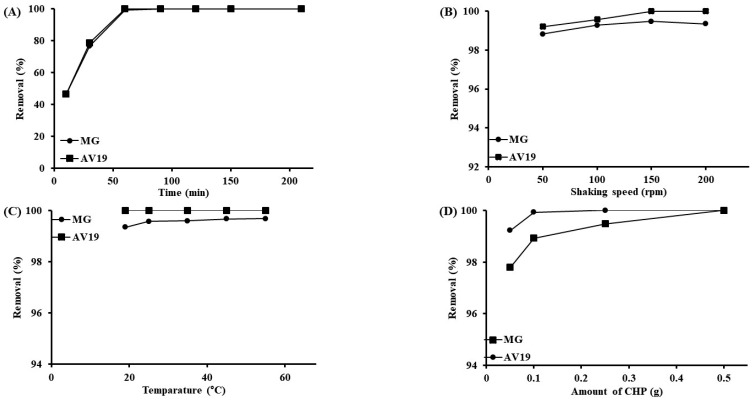
The effect of interaction time (pH 4, 100 mg/L dyes, 150 rpm shaking speed, 0.1 g CHP, 25 °C, 25 mL of dye solutions) (**A**), shaking speed (pH 4, 100 mg/L dyes, 60 min, 0.1 g CHP, 25 °C, 25 mL of dye solutions) (**B**), temperature (pH 4, 100 mg/L dyes, 60 min, 150 rpm shaking speed, 0.1 g CHP, 25 mL of dye solutions) (**C**), and amount of CHP (pH 4, 100 mg/L dyes, 60 min, 150 rpm shaking speed, 25 °C, 25 mL of dye solutions) (**D**).

Another examined parameter is the interaction time of the polymer and the dyes. Experiments were conducted using 100 mg/L dyes (25 mL) and 0.1 g of polymer at 150 rpm shaking speed and 25 °C. The dyes have shown similar behavior over time. AV19 removal was completed (~100%) in 60 min, while MG removal remained ~99% in 60 min and reached ~100% in 90 min (Figure 7A). This slight difference occurred because AV19 has more ionic groups. The ~46% removal efficiency was achieved for both dyes in the first 10 min. This rapid and high removal occurred because the dye solution quickly enters the polymer structure, and the polymer swells. Other parameters, such as shaking speed (Figure 7B), temperature (Figure 7C), and amount of polymer (Figure 7D), did not affect the removal much. Shaking speed can affect removal by changing the film thickness around the adsorbent. However, this effect was not observed in swellable polymers due to the quickly penetrating solution into the film. Removal efficiencies for both dyes are around ~99% and ~100% for all the tried shaking speeds. Similarly, removal rates at different temperatures are around ~99% and ~100% for both dyes. Temperature can increase the kinetic energy of the dyes, allowing them to move to the adsorbent. Thus, removal efficiency increases. It is also possible that the removal can decrease with temperature. The deterioration of the interaction between the adsorbent and the adsorbate with temperature reduces the removal. Neither of these effects was observed in the swellable polymer–dye interaction. When the adsorbent is used in small amounts, low removal is expected. However, when 0.05 g of polymer is used, the removal efficiency is ~98% for MG and ~99% for AV19.

**Figure 8:**
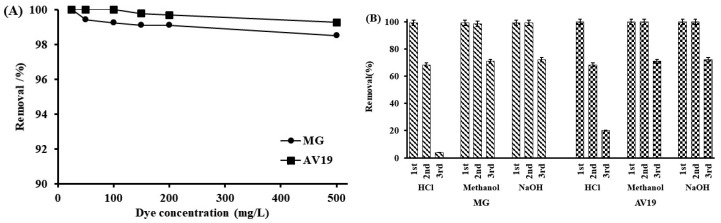
Effect of initial dye concentration on the removal (pH 4, 150 rpm shaking speed, 0.1 g CHP, 60 min, 25 °C, 25 mL of dye solutions) (**A**) and reuse of CHP (pH 4, 100 mg/L dye, 150 rpm shaking speed, 60 min, 25 °C, 25 mL of dye solutions) (**B**).

The high removal capacity of the CHP was seen more clearly when the dye concentrations were increased. Although the concentrations were increased to 500 mg/L (1000 mg/L total), the obtained removal efficiency with 0.1 g of polymer was still ~98% (Figure 8A). The reusability of the polymer is also essential. Due to its magnetic properties, the polymer can easily be taken from the solution with an external magnet, but the adsorbed dyes must be removed for reuse. The 1 M HCl, 90% methanol, and 1 M NaOH solutions were tested for polymer regeneration (Figure 8B). The removal efficiencies decreased to ~68% for both dyes in the second use after HCl regeneration. The decrease in removal efficiency increased further in the third use. The obtained removal percentages are ~4% for MG and ~20% for AV19 in the third use. The ingress of HCl into the polymer can also dissolve Fe_3_O_4_ particles. Thus, the created network was disrupted, and the polymer structure was damaged. Consequently, removal efficiencies are significantly reduced. In contrast, higher reuse values were obtained when methanol and NaOH were used for regeneration. The removal percentages are between ~98% and ~100% in the second and ~71% and ~72% in the third uses for both dyes. Methanol disrupted the dye–polymer interaction and removed the bound dye. In addition to this effect, ion exchange also occurred in NaOH. OH ions replaced the negative groups of the dyes bound to the polymer, thus removing the dyes. NaOH gave slightly better results. The decrease in the third use is due to the decline in the number of non-interacted groups on the CHP and the slight reduction in the CHP amount after each regeneration process.

### 3.3. Kinetic and Isotherm Parameters

**Figure 9:**
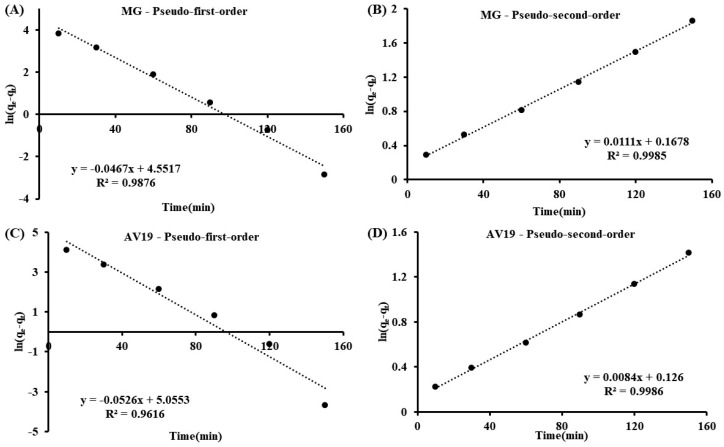
Kinetic plots of MG (**A**,**B**) and AV19 (**C**,**D**).

Pseudo-first-order Equation (2) and pseudo-second-order Equation (3) [38] parameters were calculated according to the following formulas:

(2) $$\ln\left(q_{e}-q_{t}\right)=lnq_{e}-k_{1}t$$

(3) $$\frac{t}{q_{t}}=\frac{1}{k_{2}q_{e}^{2}}+\frac{1}{q_{e}}t$$

where q_e_ and q_t_ are the removed dye amount at equilibrium in mg/g and t (min) time, k_1_ (1/min) and k_2_ (g/mg min) are rate constants. The plots are shown in Figure 9, and the calculated parameters are shown in Table 3. The pseudo-second-order kinetic model’s coefficient of determination (R^2^) is higher than the pseudo-first-order model’s. This indicates that chemisorption is a rate-limiting step.

**Figure 10:**
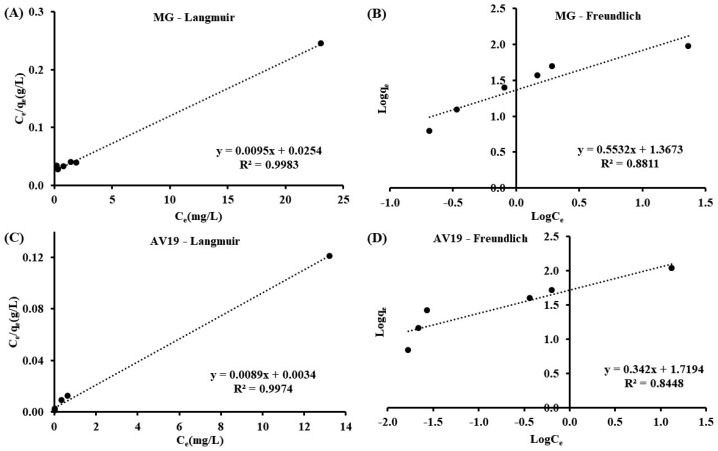
Isotherm plots of MG (**A**,**B**) and AV19 (**C**,**D**).

Langmuir Equation (4) and Freundlich Equation (5) isotherms were also applied to the method (Figure 10 and Table 3). The following formulas were used [39]:

(4) $$\frac{C_{e}}{q_{e}}=\frac{1}{Q_{m}K_{L}}+\frac{C_{e}}{Q_{m}}$$

(5) $$\;logq_{e}=logK_{f}+\frac{1}{n}logC_{e}$$

where C_e_ is the residual concentration of dye at equilibrium in mg/L, Q_m_ (mg/g) and K_f_ (mg/g) are the adsorption capacity of CHP, K_L_ (L/mg) is the adsorption energy, and n is the adsorption intensity. The R^2^ value of the Langmuir model is higher than that of the Freundlich model. The experimentally obtained maximum adsorption removals are ~94 mg/g for MG and ~109 mg/g for AV19. These values agree with the maximum adsorption capacities calculated from the Langmuir isotherm (105.33 mg/g for MG and 112.30 mg/g for AV19). The adsorption fitted the Langmuir isotherm and therefore occurred as a monolayer.

### 3.4. Comparison with Similar Studies

Table 4 compares the removal performance of the novel CHP with similar works. The removal efficiencies are generally high in polymer-based removal studies. The swollen polymer causes more interactions with the penetrated pollutants, resulting in higher removal capacities. CHP also has an acceptable removal capacity against the working textile dyes.

### 3.5. Antibacterial Activity of CHP

Textile wastewater can harbor various microorganisms due to its organic constituents, such as cellulose and natural fibers. These compounds can degrade into monomers, creating a nutrient-rich environment that promotes the growth of harmful microorganisms. Therefore, eliminating microorganisms during the dye removal process is becoming increasingly important to prevent the release of pathogenic bacteria into the environment [40]. In this context, the antibacterial activity of CHP against various pathogenic bacteria was examined. The results showed that CHP is highly effective against Gram-positive (*L. monocytogenes* and *S. aureus*) and Gram-negative (*P. aeruginosa* and *E. coli* O157:H7, *E. coli* type 1) bacteria (Figure S5). The mean diameters of the inhibition zones are shown in Table 5. The highest inhibition zone (29.55 ± 0.89) was observed against *L. monocytogenes*, while the lowest inhibition zone (16.63 ± 0.40) was recorded against *E. coli* O157:H7. Harmful species can alter the permeability of the cell membranes. Thus, they enter bacteria, damage protein functions, and bacterial inhibition occurs [41]. Also, reactive oxygen species can be formed inside the cell, causing oxidative damage to cell walls, proteins, and DNA. As a result, cells die, and bacterial growth does not occur [42]. The diameter of the inhibition zone indicates the area with no bacterial growth and is directly proportional to the antibacterial activity [43]. Different materials’ zone of inhibition values in the literature were also examined. Prameela et al. [44] reported inhibition zone diameters of Zn-doped Co_3_O_4_ against *S. aureus* and *E. coli* are 16 mm and 18 mm. Saharaf et al. [45] synthesized Ag@Fe_3_O_4_ core–shell nanoparticles and found a 28 mm diameter against *E. coli*. Abdulsada et al. [46] investigated the antibacterial activity of Fe_3_O_4_ NPs+polyethylene glycol+gentamicin composite. The stated diameters are 23.66 mm for *S. epidermidis*, 25.66 mm for *P. mirabilis*, and 26.33 mm for *A. baumannii.* CHP showed good antibacterial activity compared with the results of the literature.

## 4. Conclusions

A new magnetic composite hydrogel polymer, which consists of acrylamide, 2,4,6-triallyloxy-1,3,5-triazine, and Fe_3_O_4_, was synthesized. The composite was hydrolyzed before dye treatment to obtain a swellable polymer that the dye solution can penetrate more easily. Thus, CHP gains a high removal capacity (>100 mg/g) against textile dyes. The highest removal efficiencies (~99% for MG and ~100% for AV19) were obtained at pH 4, 150 rpm shaking speed, in 60 min, 25 °C, using 100 mg/L of each dye (25 mL) and 0.1 g CHP. The derivative method was developed to calculate the concentration of dyes in the mixture individually. The accuracy and precision values of the method are at an acceptable level. The removal procedure fits with pseudo-second-order kinetics and Langmuir isotherm models. CHP has great antibacterial activity against both Gram-positive and Gram-negative bacteria.

## Figures and Tables

**Table 1:** LOD, LOQ, and calibration equations.

Dye	λ (nm)	Calibration Equations	R^2^	LOD (mg/L)	LOQ (mg/L)
AV19	428.0	A = 0.0007C_AV19_ − 0.0002	0.9996	0.0754	0.2283
MG	545.5	A = 0.0009C_MG_ − 0.0001	1.0000	0.0305	0.0924

**Table 2:** R% and RSD% values of the dye mixture solutions.

Mixture	Added (mg/L)		Found (mg/L)		Recovery (%)	
	AV19	MG	AV19	MG	AV19	MG
1	3.00	1.00	3.13	0.98	104.33	98.00
2	5.00	1.00	5.11	0.96	102.20	96.00
3	7.00	1.00	7.14	0.98	102.00	98.00
4	10.00	1.00	10.28	1.03	102.80	103.00
5	1.00	3.00	1.03	2.98	103.00	99.33
6	5.00	3.00	5.21	2.92	104.20	97.33
7	7.00	3.00	7.24	3.09	103.43	103.00
8	10.00	3.00	10.26	3.02	102.60	100.67
9	1.00	5.00	1.00	5.02	100.00	100.40
10	3.00	5.00	2.88	4.94	96.00	98.80
11	7.00	5.00	7.19	5.20	102.71	104.00
12	10.00	5.00	10.34	5.18	103.40	103.60
13	1.00	7.00	0.98	6.90	98.00	98.57
14	3.00	7.00	2.90	6.92	96.67	98.86
15	5.00	7.00	5.03	7.11	100.60	101.57
16	10.00	7.00	10.16	7.37	101.60	105.29
17	1.00	10.00	0.98	10.03	98.00	100.30
18	3.00	10.00	2.88	10.12	96.00	101.20
19	5.00	10.00	4.91	10.18	98.20	101.80
20	7.00	10.00	6.91	10.22	98.71	102.20
21	1.00	1.00	1.02	0.98	102.00	98.00
22	3.00	3.00	3.03	2.95	101.00	98.33
23	5.00	5.00	5.08	5.02	101.60	100.40
24	7.00	7.00	7.09	7.20	101.29	102.86
25	10.00	10.00	10.06	10.41	100.60	104.10
			Mean		100.84	100.62
			SD		2.45	2.42
			RSD%		2.43	2.40

**Table 3:** Kinetic and isotherm parameters.

Parameters		MG	AV19
Pseudo-first-order kinetic	q_e_ (mg/g)	94.80	156.85
	k_1_ (1/min)	0.0467	0.0526
	R^2^	0.9876	0.9616
Pseudo-second-order kinetic	q_e_ (mg/g)	90.17	118.36
	k_2_ (1/min)	0.0007	0.0006
	R^2^	0.9985	0.9986
Langmuir isotherm	Q_m_ (mg/g)	105.33	112.30
	K_L_ (L/mg)	0.37	2.66
	R^2^	0.9983	0.9974
Freundlich isotherm	K_f_ (mg/g)	23.30	52.41
	n	1.81	2.92
	R^2^	0.8811	0.8448

**Table 4:** Comparison of the obtained results with similar studies.

Material	Adsorbate pH	Removal Efficiency	Calculated Adsorption Capacity	Ref.
Fe_3_O_4_/hyper-cross-linked polymer composites	Crystal Violet/pH 7	94.1%	Q_m_ = 104.3 mg/g K_F_ = 58.431 (mg/g)·(L/mg)^1/n^	[16]
Graphene/polyaniline/ Fe_3_O_4_	Congo Red pH 6.3	92.4%	Q_m_ = 248.76 mg/g K_F_ = 82.41	[17]
Fe_3_O_4_/ polypyrrole/carbon black	Congo Red/pH 7 Methylene Blue/pH 8	96.9% 95.9%	Q_m_ = 500 mg/g K_F_ = 120 L/mg Q_m_ = 90.9 mg/g K_F_ = 89.2 L/mg	[18]
Fe_3_O_4_/poly(2-hydroxyethylmethacrylateco- 2-acrylamido-2-methylpropanesulfonic acid)	Methylene Blue/pH 6.5	99.01%	Q_m_ = 445.35 mg/g K_F_ = 95.59 mg/g	[19]
Fe_3_O_4_ embedded chitosan–crosslinked-polyacrylamide	Sunset yellow pH 2–10	>99%	Q_m_ = 359.71 mg/g K_f_ = 33.21 mg^1- (1/n)^ L^1/n^ g^−1^	[20]
Fe_3_O_4_ embedded acrylamide- 1,3,5-Triacryloylhexahydro-1,3,5-triazine polymer	Disperse Blue 56 pH 2	96.6%	Q_m_ = 0.9063 mg/g K_F_ = 1.2735 mg/g	[21]
Fe_3_O_4_/Acrylamide/2,4,6-triallyloxy-1,3,5-triazine composite hydrogel polymer	Malachite Green/pH 4 Acid Violet 19/pH 4	~99% ~100%	Q_m_ = 105.33 mg/g K_F_ = 23.30 mg/g Q_m_ = 112.30 mg/g K_F_ = 52.41 mg/g	This study

**Table 5:** Antibacterial activity of CHP.

Bacteria	Zone of Inhibition (mm)
*Pseudomonas aeruginosa*	22.58 ± 0.68
*Escherichia coli* O157:H7	16.63 ± 0.40
*Escherichia coli* type 1	22.48 ± 0.63
*Staphylococcus aureus*	23.53 ± 0.55
*Listeria monocytogenes*	29.55 ± 0.89

## Data Availability

The original contributions presented in this study are included in the article/Supplementary Materials. Further inquiries can be directed to the corresponding author.

## Supplementary Materials

The following supporting information can be downloaded at: https://www.mdpi.com/article/10.3390/polym17182469/s1, Figure S1: Calibration graphs of AV19 (a) and MG (b); Figure S2: Absorption (a) and first derivative spectra (b) of dye mixtures; Figure S3: Photograph of CHP; Figure S4: Magnetic behavior of the CHP; Figure S5: Antibacterial activity of CHP against *Pseudomonas aeruginosa* (a), *Escherichia coli O157:H7* (b), *Escherichia coli* type 1 (c), *Staphylococcus aureus* (d), and *Listeria monocytogenes* (e).
