# Supplementary material for: Removal of Textile Dye Mixture by Fe3O4/Acrylamide/Triacryloylhexahydro Triazine Composite Hydrogel Polymer
Source: Polymers (Basel). 2025 Sep 12;17(18):2469. doi: 10.3390/polym17182469 (PMC12473609; doi:10.3390/polym17182469)
Supplement: Supplementary file 1 [file polymers-17-02469-s001.zip › polymers-3821926-supplementary.pdf]

polymers

MDPI

Article

Removal of Textile Dye Mixture by Fe_3O_4/Acrylamide/Triacryloylhexahydro Triazine Composite Hydrogel Polymer

Sude Sena Erdağı [1], Can Serkan Keskin [2,*], Semra Yılmazer Keskin [2] and Ayşe Avcı [3]

[1] Institute of Natural Science, Sakarya University, Sakarya 54050, Türkiye; sena.erdagi@ogr.sakarya.edu.tr
[2] Faculty of Science, Department of Chemistry, Sakarya University, Sakarya 54050, Türkiye; syilmazer@sakarya.edu.tr
[3] Faculty of Engineering, Department of Food Engineering, Sakarya University, Sakarya 54050, Türkiye; aysea@sakarya.edu.tr
* Correspondence: ckeskin@sakarya.edu.tr; Tel.: +90-2642956036

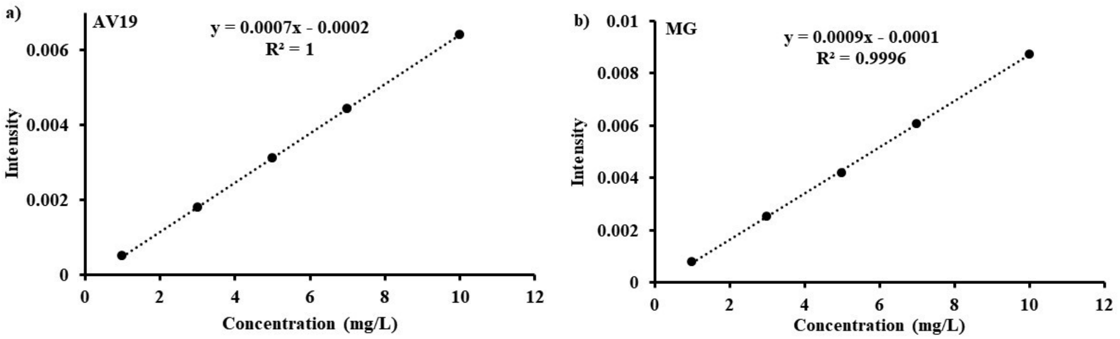

Figure S1. Calibration graphs of AV19 (a) and MG (b) drawn in the concentration range of 1.0 – 10 mg/L.

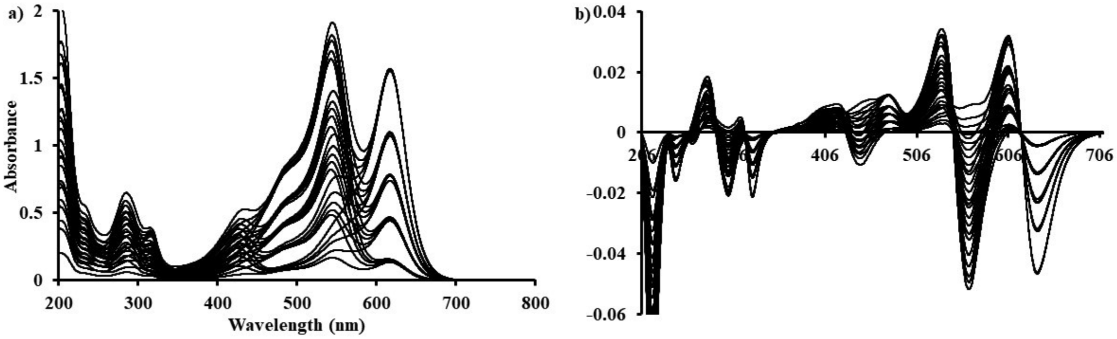

Figure S2. Absorption (a) and first derivative spectra (b) of dye mixtures.

https://doi.org/10.3390/xxxxx

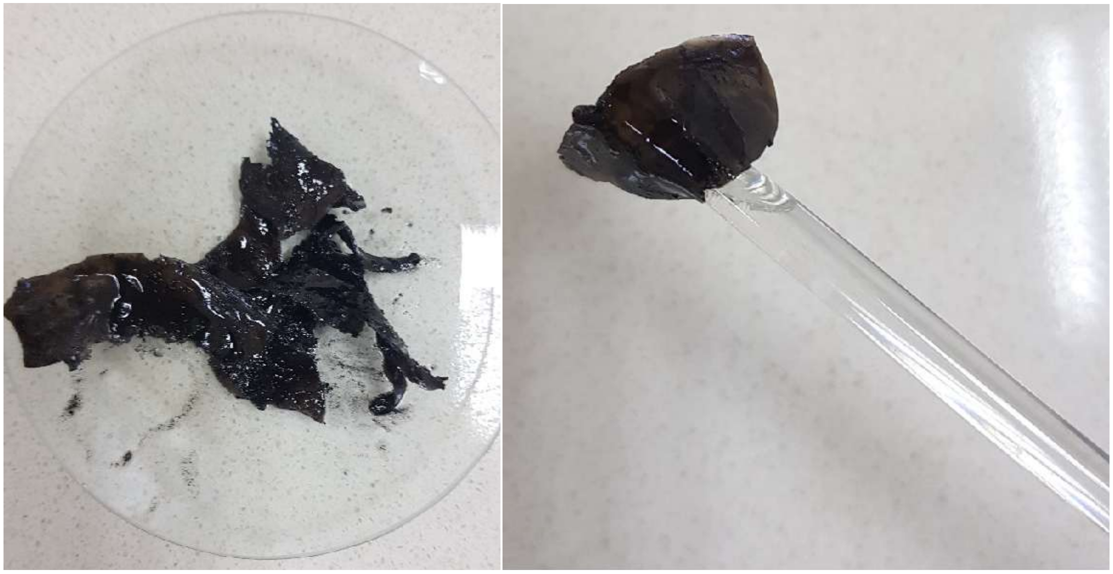

Figure S3. Photograph of CHP.

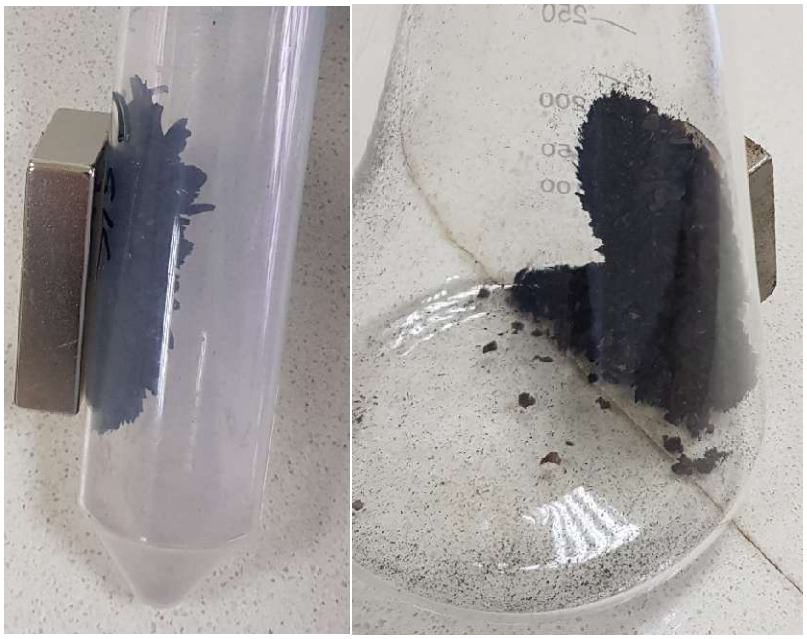

Figure S4. Magnetic behavior of the CHP.

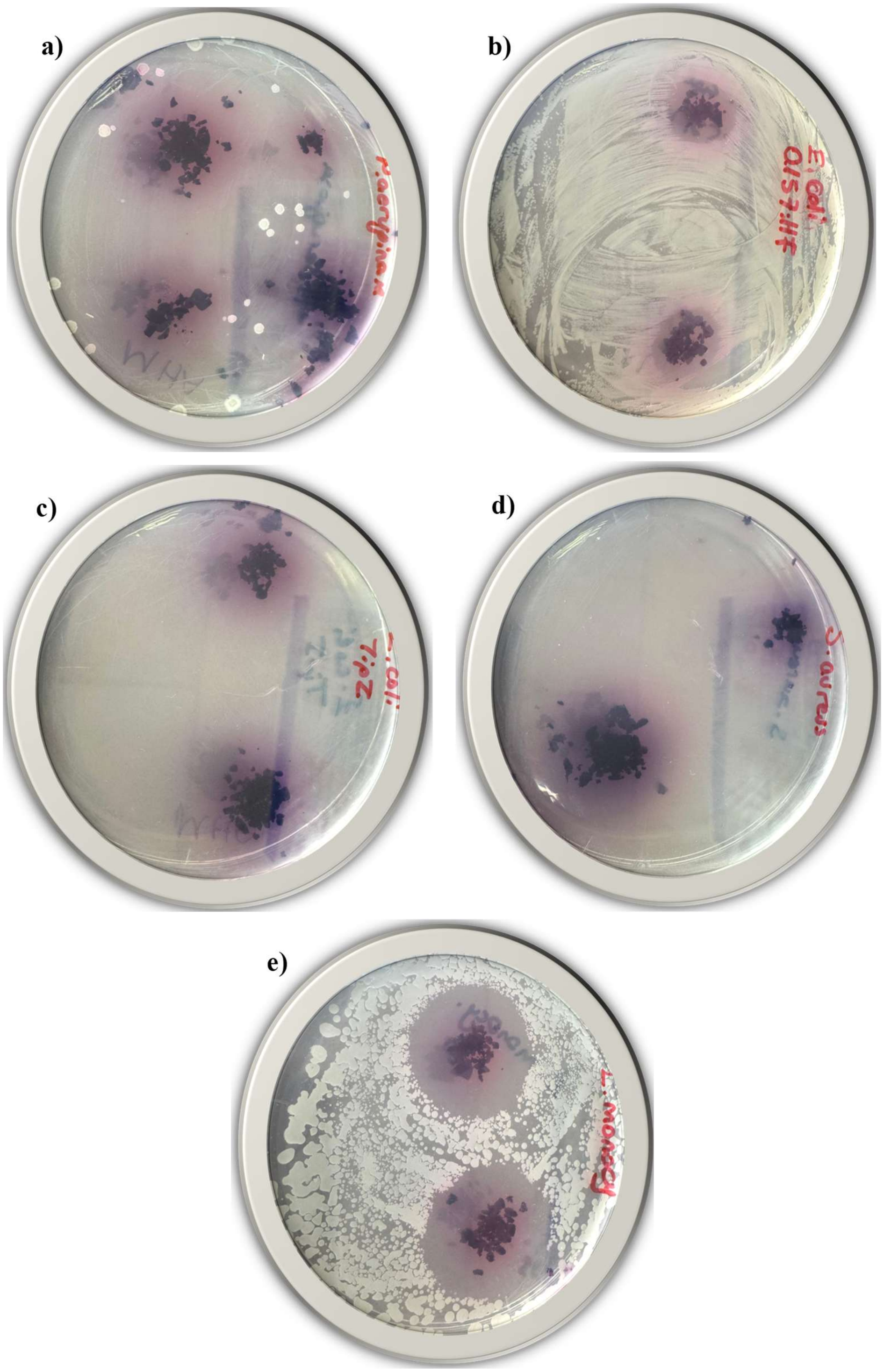

Figure S5. Antibacterial activity of CHP against Pseudomonas aeruginosa (a), Escherichia coli O157:H7 (b), Escherichia coli type 1 (c), Staphylococcus aureus (d), and Listeria monocytogenes (e).
